# Asian American Older Adults and Social Isolation: A Systematic Literature Review

**DOI:** 10.1093/geroni/igaa057.1051

**Published:** 2020-12-16

**Authors:** Simona Kwon, Deborah Min, Stella Chong

**Affiliations:** NYU School of Medicine, New York, New York, United States

## Abstract

Asian Americans are the fastest growing racial and ethnic minority group in the United States, whose population is aging considerably. Previous studies indicate that social isolation and loneliness disproportionately affects older adults and predicts greater physical, mental, and cognitive decline. A systematic literature review using PRISMA guidelines was conducted to address this emerging need to understand the scope of research focused on social isolation and loneliness among the disparity population of older Asian Americans. Four interdisciplinary databases were searched: PubMed, CINAHL, PsycINFO, and AgeLine; search terms included variations on social isolation, loneliness, Asian Americans, and older adults. Articles were reviewed based on six eligibility criteria: (1) research topic relevance, (2) study participants aged >60 years, (3) Asian immigrants as main participants, (4) conducted in the United States, (5) published between 1995-2019, and (6) printed in the English language. The search yielded 799 articles across the four databases and 61 duplicate articles were removed. Abstracts were screened for the 738 remaining studies, 107 of which underwent full-text review. A total of 56 articles met the eligibility criteria. Synthesis of our review indicates that existing research focuses heavily on Chinese and Korean American immigrant communities, despite the heterogeneity of the diverse Asian American population. Studies were largely observational and employed community-based sampling. Critical literature gaps exist surrounding social isolation and loneliness in Asian American older adults, including the lack of studies on South Asian populations. Future studies should prioritize health promotion intervention research and focus on diverse understudied Asian subgroups.

